# Conserved Regulatory DNA Methylation in Human and MYC/PI3K-Driven Murine EBV-Negative Burkitt Lymphoma

**DOI:** 10.3390/cancers18203408

**Published:** 2026-10-21

**Authors:** Selina Glaser, Meiqi Ren, Sandrine Sander, Reiner Siebert

**Affiliations:** 1 Institute of Human Genetics, Ulm University and Ulm University Medical Center, 89081 Ulm, Germany; 2 German Cancer Research Center (DKFZ) Heidelberg, Division Adaptive Immunity and Lymphoma, 69120 Heidelberg, Germany; 3 Comprehensive Cancer Center (CCC) Mecklenburg-Vorpommern, University Medicine Greifswald, 17489 Greifswald, Germany; 4 German Center for Child and Adolescent Health (DZKJ), Partner Site Ulm, 89081 Ulm, Germany; 5 Center for Rare Diseases (ZSE) Ulm, Ulm University and Ulm University Medical Center, 89081 Ulm, Germany

**Keywords:** Burkitt lymphoma, DNA methylation, transgenic mouse models

## Abstract

**Background/Objectives:** Burkitt lymphoma (BL) is an aggressive germinal center B-cell (gcBC) malignancy driven by *MYC* deregulation and cooperating molecular alterations. A mouse model combining constitutive MYC and PI3K activity in gcBC closely recapitulates key features of human BL, but its epigenetic similarity to the human disease has not been investigated in detail. Here, we compared genome-wide DNA methylation patterns between human sporadic EBV-negative BL and this murine model. **Methods:** DNA methylation profiles of 37 human BL samples and 20 gcBC controls were compared with those of 8 murine tumors from the MYC/PI3K driven mouse model and 11 murine gcBC controls using Illumina methylation arrays. Differentially methylated CpGs were identified using linear modeling, and chromatin-state enrichment, cross-species gene overlap, protein interaction, and pathway enrichment analyses were performed. **Results:** BL showed global DNA hypomethylation in both species and distinct methylation profiles from gcBCs. CpGs hypomethylated in BL were enriched at enhancers, whereas hypermethylated CpGs were strongly enriched at poised promoters. We identified 695 genes with shared hypermethylation and 512 with shared hypomethylation between human and murine BL. Restricting the analysis to regulatory regions identified 86 genes consistently hypomethylated in both species. These genes were significantly enriched for regulation of intracellular signal transduction and sphingolipid signaling. Included are regulators of GTPase and kinase signaling, such as *GNA13*, *RASA3*, *RASAL1*, *SPRED2*, *PIK3R2*, *PRKCA*, *PRKCE*, and *FYN*. The conserved set also contained genes with established roles in germinal center B-cell biology, including *FOXO1*, *BACH2*, and *NR3C1*. **Conclusions:** Human and murine BL share conserved regulatory DNA methylation features, supporting the MYC/PI3K mouse model as a system to study epigenetic mechanisms of BL.

## Simple Summary

Burkitt lymphoma is a fast-growing blood cancer driven by abnormal activation of the *MYC* gene. Mouse models are widely used to study its development, but their similarity to human disease needs to be established. Here, we compared DNA methylation, an important regulator of gene activity, between human EBV-negative Burkitt lymphoma and a mouse model driven by *MYC* and phosphoinositide 3-kinase activation specifically in germinal center B-cells. We found similar methylation patterns in both species, particularly in regulatory regions of genes involved in cell signaling and B-cell biology. These findings show that this mouse model reproduces important epigenetic features of human Burkitt lymphoma and can help investigate conserved mechanisms of the disease.

## 1. Introduction

Burkitt lymphoma (BL) is a highly aggressive B-cell malignancy in which the tumor cells resemble germinal center B-cells (gcBCs) [1,2,3]. It is characterized by deregulation of the *MYC* oncogene and a very high proliferative rate. *MYC* is typically activated in BL by chromosomal translocation to an immunoglobulin locus, resulting in sustained MYC expression and extensive changes in proliferation, metabolism, and cell survival [2]. Al-though *MYC* deregulation is a defining feature of BL, *MYC* activation alone is insufficient to induce lymphoma, highlighting the importance of cooperating oncogenic pathways and genetic alterations [4,5,6,7,8,9,10]. These recurrent alterations affect several key pathways, including the ID3/TCF3 axis and tonic B-cell receptor signaling, the GNA13/RHOA/P2RY8 pathway involved in gcBC positioning, cell-cycle regulation via CCND3, apoptosis and tumor suppression via TP53, and chromatin remodeling through SMARCA4 and other members of the SWI/SNF complex [4,5,6,7,8,9,10,11]. Many of these alterations converge on pathways that promote cell survival and proliferation, most notably tonic B-cell receptor and PI3K signaling.

Epidemiologic studies differentiate three subtypes of BL: endemic, sporadic and immunodeficiency-associated BL [1,3,12]. These subtypes of BL are intimately linked to the presence of Epstein–Barr Virus (EBV) in the tumor cells, with endemic BL being in >90% of cases EBV-positive and sporadic BL being in the vast majority EBV-negative [2]. Remarkably, EBV-negative BL show a higher frequency of somatic mutations in genes commonly altered in BL and a lower frequency of aberrant somatic hypermutation than EBV-positive BL [9,13,14]. In turn, DNA methylation, an epigenetic mechanism contributing to the biology of B-cell malignancies, has been shown to affect regulatory sites of such driver genes predominantly in EBV-positive BL [15].

DNA methylation is an important epigenetic mechanism that regulates gene expression and chromatin activity and plays a central role in normal B-cell differentiation [16]. In BL, genome-wide DNA methylation studies using whole-genome bisulfite sequencing and array-based approaches have identified widespread and biologically relevant methylation alterations [11,15,17]. Kretzmer et al., by combining various sequencing strategies, demonstrated that BL-associated differentially methylated regions are linked to transcriptional regulation and genes recurrently somatically mutated, highlighting an interplay between genetic and epigenetic alterations [11]. More recently, array-based studies have identified distinct DNA methylation patterns in BL that are associated with EBV status as well as molecular and clinical characteristics [15,17]. In particular, EBV-negative BL showed a relatively lower methylated profile, with hypomethylated regions enriched in pathways related to B-cell receptor signaling. In contrast, hypermethylation was observed in EBV-positive BL and preferentially affected Polycomb-associated regions and regulatory elements of genes frequently mutated in BL, including *CCND3* and *TP53* [15]. Both Thomas et al. and Glaser et al. further identified two major BL epitypes, named HypoBL and HyperBL or BL-mC1/2 and BL-mC3/4, characterized by differences in global DNA methylation [15,17].

Mouse models are widely used to study cancer development in general and lymphomagenesis in particular. Though their epigenetic similarity to human disease needs to be investigated, they offer in principle the option of identifying pathways and modules epigenetically altered across species [18,19].

Regarding murine models for MYC activation, a whole range has been established giving rise to various kinds of tumors and reflecting the key role of MYC in tumor initiation and progression [20,21,22]. Depending on the cellular compartment of aberrant MYC expression, these can also give rise to different lymphoma phenotypes, highlighting the importance of both the cell of origin and cooperating oncogenic events in determining tumor development and characteristics [23,24].

In 2012, a genetically engineered mouse model was described by Sander et al. resulting in lymphomas that closely recapitulate human EBV-negative BL [25]. This mouse model combined MYC overexpression with constitutive activation of the phosphoinositide 3-kinase (PI3K) pathway specifically in gcBCs. Thus, it resembled key features of supposedly early events in human EBV-negative BL. The histological appearance, surface marker expression, germinal center phenotype, and gene expression profile of the tumors well aligned with that for human EBV-negative BL. Finally, the tumors also acquired additional genetic alterations, including alterations affecting genes like *Ccnd3* and *Foxo1* recurrently mutated in human BL [25,26]. This model therefore provides a well-established and probably best murine system for studying the molecular mechanisms of MYC-driven EBV-negative BL and the cooperation between MYC and PI3K signaling.

Although the DNA methylation landscape of human EBV-negative BL has meanwhile been comprehensively described, a systematic comparison of BL-associated DNA methylation changes between human BL and the MYC/PI3K-driven mouse model has not been performed. In particular, it remains unclear whether conserved DNA methylation changes preferentially affect functional regulatory regions and specific biological pathways.

Here, we compared established genome-wide DNA methylation profiles of primary human EBV-negative (sporadic) BL with those of tumor cells isolated from the MYC/PI3K-driven transgenic mouse model developed by Sander et al. [25], using human and murine gcBCs as reference populations.

## 2. Materials and Methods

### 2.1. Human Lymphoma and Control Samples

The human lymphoma cohort comprised 37 EBV-negative sporadic BL cases previously described by Glaser et al. [15]. As outlined previously [15], the cases were reviewed by an expert hematopathology panel and selected based on a high tumor cell content (>60%), presence of an *IG*::*MYC* translocation confirmed by fluorescence in situ hybridization or whole-genome sequencing, and absence of *BCL2* and *BCL6* rearrangements. The cohort included tumors of 32 pediatric patients (≤18 years) and 5 adults (>18 years), with a median age at diagnosis of 10 years (range, 2–44 years). The majority of tumor samples (33/37) were cryopreserved, while 4 samples were formalin-fixed, paraffin-embedded. As a non-malignant reference cohort, 20 gcBC samples were included [11,16,27] (Supplementary Table S1). Samples were collected and analyzed within the studies of the “Molecular Mechanisms in Malignant Lymphoma (MMML)” network and the ICGC MMML-Seq network [15].

### 2.2. Murine Lymphoma and Control Samples

The murine cohort comprised cryopreserved samples from 8 MYC/PI3K-driven lymphomas and 11 non-transformed control populations from the genetically engineered mouse model previously described by Sander et al. (Supplementary Table S2) [25]. Details on the different populations as well as sorting strategies are given in Supplementary Table S2. The animal care and experimental procedures involving mice were approved by the internal animal welfare committees of the respective institutions as well as by state regulatory authorities (Regierungspräsidium Karlsruhe G-119/17; G-120/19; Landesamt für Gesundheit und Soziales [LaGeSo] Berlin G0374/13).

### 2.3. DNA Methylation Profiling and Preprocessing

For human samples, previously published DNA methylation data generated using the Infinium HumanMethylation450 BeadChip (450K, Illumina Inc., San Diego, CA, USA) were analyzed [11,15]. DNA methylation data were processed and quality-controlled as described previously [28]. DNA methylation data were normalized using the minfi package (v1.54.1) with an Illumina-like normalization approach based on the intrinsic array control probes and without background correction [29]. Beta values, representing the percentage of DNA methylation at each CpG site, were calculated from the normalized signal intensities. For quality control and filtering, probes targeting rs loci or gonosomal loci were excluded, as well as probes with a detection *p*-value > 0.01, resulting in 473,864 CpG sites.

For murine samples, DNA methylation profiling was performed as custom service by Life & Brain GmbH (Bonn, Germany) using the Infinium Mouse Methylation BeadChip (Illumina Inc., San Diego, CA, USA). Raw intensity data were normalized using GenomeStudio (v2011.1; Methylation Module v1.9.0; Illumina Inc., San Diego, CA, USA) with default settings and internal normalization controls, resulting in 266,961 CpG sites. Quality control and filtering of the murine data were performed using criteria complementary to those applied to the human data.

### 2.4. Chromatin State Mapping

Chromatin states in human gcBCs were based on the data from Hübschmann et al., with raw sequencing data publicly available from the Blueprint DCC portal [11,30].

The murine ChIP-seq data used for chromatin state segmentation were previously generated and analyzed by Glaser et al. [18]. Data are available ChIP-Seq data for H3K27ac, H3K27me3, H3K4me1, H3K4me3, and H3K36me3 marks from spleen and thymus of 8-week-old mice were sourced from ENCODE (Lab: Bing Ren, UCSD). Using these data, the mouse genome was segmented into a ten-state combinatorial chromatin model and then collapsed into five functional chromatin states (heterochromatin, poised promoter, promoter, enhancer and transcription), summarizing similar states based on emission probabilities as described in Hübschmann et al. [30]. Segmentation using the ChIP-Seq data for the five histone modifications was done with the ChromHMM software (version 1.24).

### 2.5. DNA Methylation Analysis

Differentially methylated CpGs (DMCs) were identified using limma (version 3.64.3) on M-values derived from normalized beta values. To ensure both statistical significance and biological relevance, CpGs with an FDR < 0.01 and an absolute mean methylation difference (|Δβ|) > 0.30 were considered as differentially methylated [31].

For functional network analysis, shared genes associated with conserved hypomethylated CpGs in human and murine tumors were analyzed using the web-based STRING analysis tool (https://string-db.org/). Protein–protein interaction networks were generated using the default STRING settings. Genes were subsequently assigned to functional categories based on enriched biological processes and functional associations identified within STRING.

Functional enrichment analysis was performed using the web-based Enrichr platform (https://maayanlab.cloud/Enrichr/, accessed on 2 September 2026). Gene Ontology Biological Process (GO BP) and KEGG 2026 pathway enrichment analyses were conducted using as background all genes represented on both the human and murine methylation arrays after mapping murine genes to their human orthologs. Terms with an adjusted *p*-value < 0.05 were considered significantly enriched.

### 2.6. Bioinformatic and Statistical Analysis

All bioinformatic and statistical analyses were performed in R (version 4.5.1), unless otherwise specified. Copy-number alterations were assessed using conumee2 (version 2.1.2) [32], with gcBCs used as the reference set. GenVisR (version 1.39.0) was used to generate variant frequency plots [33]. Gene orthologs between human and mouse were identified using the biomaRt package (version 2.64.0) [34]. Enrichment analyses based on Fisher’s exact tests were used to assess the overlap of differentially methylated genes between species and the enrichment of differentially methylated CpGs across chromatin states. For these analyses, odds ratios (ORs) and corresponding 95% confidence intervals (CIs) were reported as measures of effect size. Unless otherwise stated, statistical tests were two-sided, and *p*-values were adjusted for multiple testing using the Benjamini–Hochberg method.

## 3. Results

**Figure 1:**
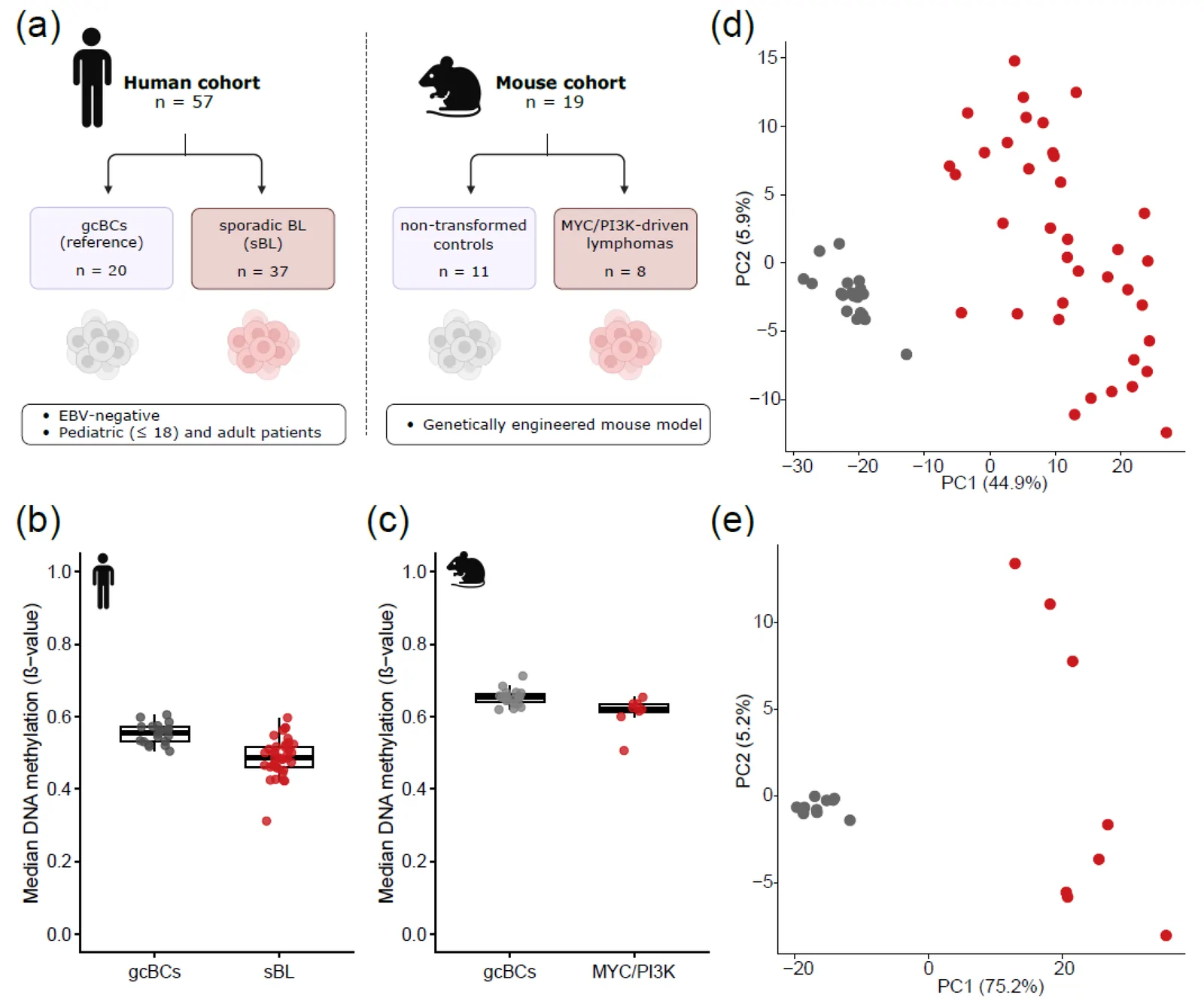
Study design and global DNA methylation landscape of human and murine Burkitt lymphoma. (**a**) Overview of the human and murine study cohorts. Schematic illustration was created with BioRender (Created in BioRender. Glaser, S. (2026) https://BioRender.com/w3en7bv, accessed on 9 October 2026). (**b**) Global DNA methylation levels in human EBV-negative sporadic Burkitt lymphoma (sBL) and germinal center B-cells (gcBCs), assessed as the median beta-value across all analyzed CpG sites. (**c**) Global DNA methylation levels in murine MYC/PI3K-driven BL and murine gcBCs. (**d**) Principal component analysis (PCA) of human samples based on the 10,000 most variable CpGs. (**e**) PCA of murine samples based on the 10,000 most variable CpGs.

To compare the DNA methylation landscape in primary human EBV-negative BL and BL from MYC/PI3K-driven transgenic mice, we performed array-based methylome profiling using Illumina Infinium BeadChip technology, an established and independently validated approach for genome-wide DNA methylation profiling [35,36]. The human cohort included previously published data of 37 primary EBV-negative BL cases and 20 gcBCs as controls. The murine cohort included 8 tumors from a MYC/PI3K-driven transgenic mouse model and 11 murine gcBCs (Figure 1a). Following normalization and quality control, a total of 473,864 CpG sites in the human dataset and 266,961 CpG sites in the murine dataset were retained for downstream analysis.

### 3.1. Human and Murine EBV-Negative BL Exhibit Distinct DNA Methylation Landscapes

Both human and murine EBV-negative BL samples showed reduced global DNA methylation compared with the respective gcBCs from the same species (Figure 1b,c), though the absolute values are not directly comparable due to the different design and content of the array. In human samples, median DNA methylation was 0.49 in BL compared with 0.57 in gcBCs (Wilcoxon rank-sum test, *p* < 0.01), while murine BL samples showed a median DNA methylation of 0.62 compared with 0.65 in control B cells (*p* < 0.01).

PCA based on the 10,000 most variable CpGs revealed clear separation of BL and control samples in both species (Figure 1d,e), indicating distinct DNA methylation profiles associated with the malignant phenotype. Annotation of the murine PCA by tissue source suggested some degree of clustering according to the anatomical origin of the tumors, although no clear separation was observed (Supplementary Figure S1). However, the small number of tumors from each tissue of origin (spleen, n = 1; mesenteric lymph nodes, n = 3; Peyer’s patches, n = 4) limits the interpretation of these patterns, and a contribution of tissue-specific effects cannot be excluded.

To further assess genomic alterations, copy number profiles were inferred from the DNA methylation array data. Human EBV-negative BL samples displayed recurrent chromosomal gains and losses previously described in EBV-negative BL, including gains affecting 1q, 7q, and 13q and losses of 6q (Supplementary Figure S2) [37]. In the MYC/PI3K transgenic mouse model, CNV analysis consistently identified trisomy of chromosome 6, a recurrent alteration with synonymous regions in human 7q and 12p previously reported in this model and proposed to contribute to lymphomagenesis (Supplementary Figure S2) [25].

### 3.2. Shared Chromatin-State Patterns of BL-Associated DNA Methylation Changes

**Figure 2:**
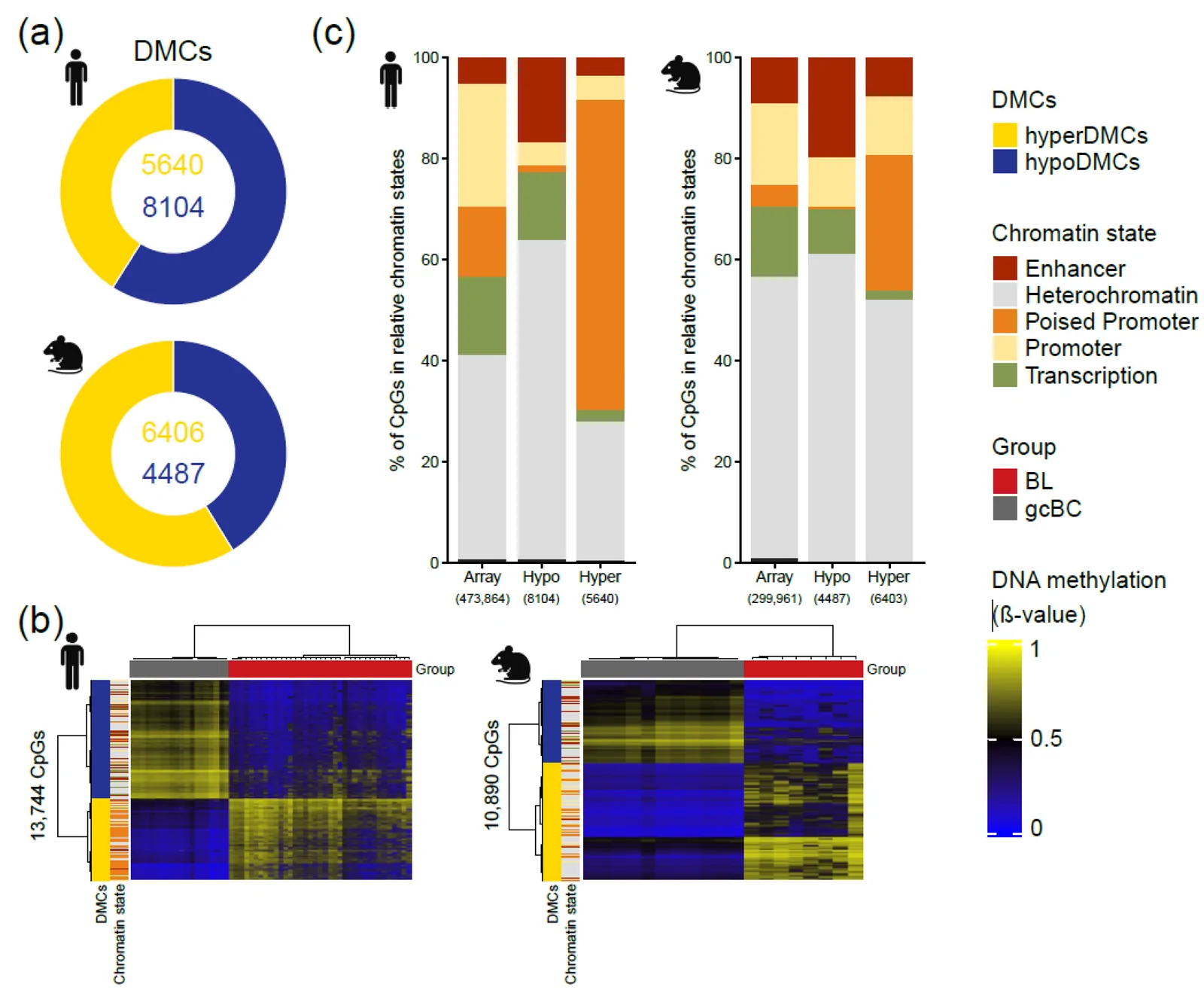
EBV-negative BL-associated DNA methylation alterations in human and murine tumors. (**a**) Number of differentially methylated CpGs (DMCs) identified in human and murine BL, classified as hypermethylated or hypomethylated according to the direction of the mean methylation difference. (**b**) Heatmaps showing DNA methylation levels of the identified DMCs across human and murine BL and their respective control samples. Differentially methylated CpGs were defined by an FDR < 0.01 and an absolute mean methylation difference (|Δβ|) > 0.3. (**c**) Distribution of hyper- and hypomethylated DMCs across chromatin states defined in germinal center B-cells (gcBCs, human) and spleen (mouse), compared with the corresponding array background.

Next, we performed differential methylation analysis in both the human EBV-negative BL and the MYC/PI3K-driven mouse model as compared to the respective gcBC populations. Considering the different content and design of the arrays as well as the slightly different sizes of the analyzed cohorts, we decided to hold FDR (<0.01) and absolute mean methylation difference (|Δβ| > 0.3) constant to allow some comparison across the analyses in both species. Using this approach, we identified extensive DNA methylation alterations in both human and murine BL as compared to the respective gcBCs. In human EBV-negative BL, 13,744 CpGs were differentially methylated as compared to human gcBC, including 8104 hypomethylated and 5640 hypermethylated CpGs (Supplementary Table S3). Similarly, 10,890 CpGs were differentially methylated in the mouse tumors as compared to the murine gcBC, comprising 4487 hypomethylated and 6403 hypermethylated CpGs (Supplementary Table S4, Figure 2a,b).

Despite differences in the overall distribution of differential CpGs between the species, both showed a similar association with chromatin states assessed from gcBCs (human) and spleen (mouse). Hypomethylated CpGs were enriched at enhancers and in heterochromatin, with stronger enrichment at enhancers in both human (OR = 3.67, 95% CI: 3.46–3.90, FDR < 0.001) and mouse (OR = 2.43, 95% CI: 2.25–2.62, FDR < 0.001) BL. Hypermethylated CpGs were strongly enriched at poised promoters in both species (human, OR = 9.95, 95% CI: 9.42–10.51; mouse, OR = 7.68, 95% CI: 7.24–8.13, FDR < 0.001) (Figure 2c).

Together, these findings indicate that BL-associated DNA methylation changes are not randomly distributed across the genome but preferentially occur within functionally distinct regulatory chromatin states, with loss of DNA methylation preferentially affecting enhancers and gain of DNA methylation preferentially affecting poised promoters in both species.

### 3.3. Conserved DNA Methylation Alterations Between Human and Murine EBV-Negative BL

**Figure 3:**
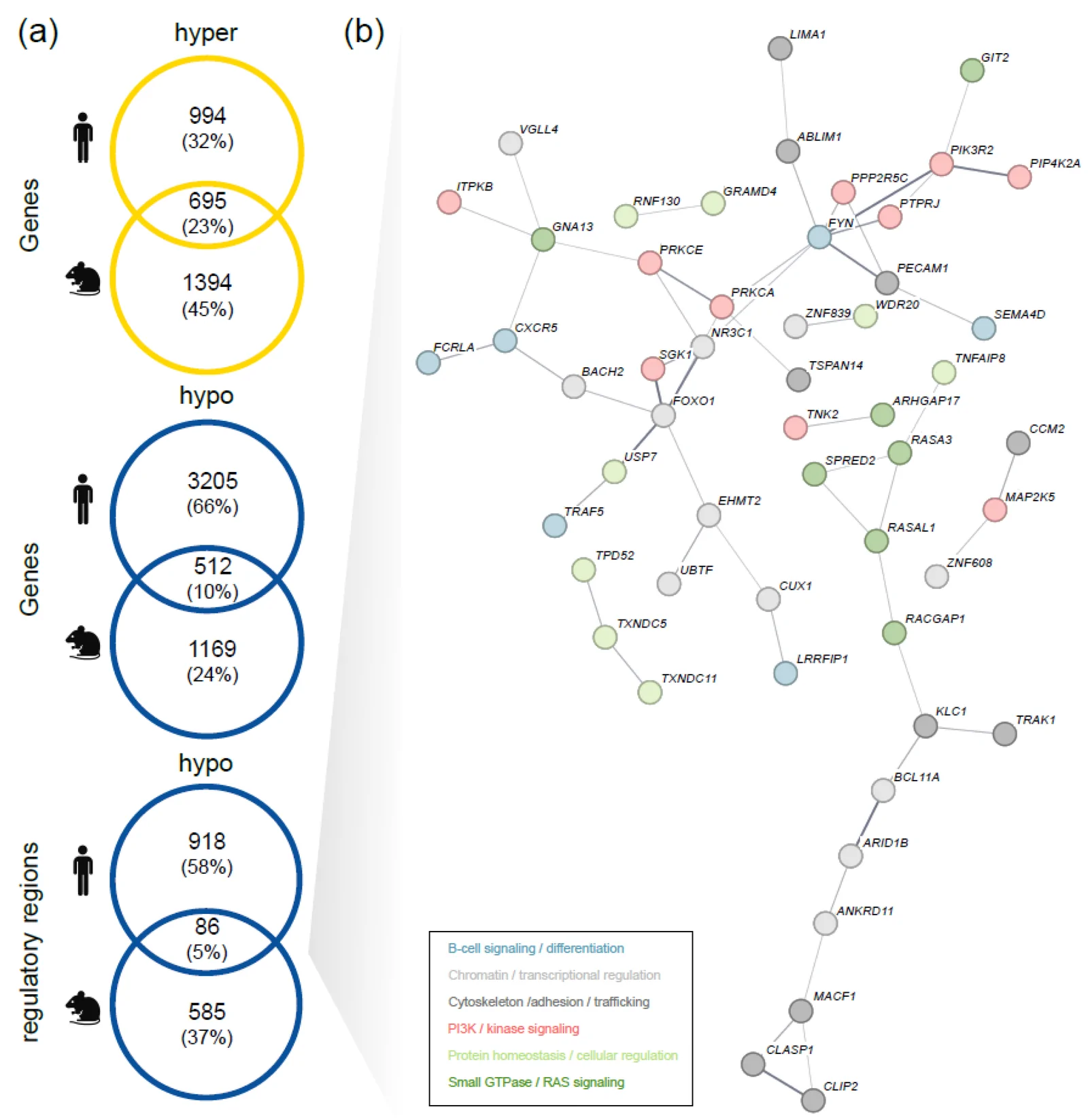
Cross-species overlap and functional characterization of differentially methylated genes. (**a**) Venn diagrams showing the overlap of genes affected by differential DNA methylation between human Burkitt lymphoma and the murine MYC/PI3K model, separately for hypomethylated (blue) and hypermethylated (yellow) genes, and the overlap of genes affected by hypomethylation restricted to regulatory regions (promoters and enhancers). (**b**) STRING network analysis of the 86 genes overlapping between humans and mice among genes hypomethylated within regulatory regions.

To determine whether EBV-negative BL-associated methylation changes were conserved across species, we compared the genes associated with differentially methylated CpGs after mapping murine genes to their human orthologs. We identified 695 shared hypermethylated genes and 512 shared hypomethylated genes (Supplementary Table S5, Figure 3a). Both overlaps were significantly greater than expected by chance (hypermethylated: OR = 9.39, 95% CI: 8.31–10.61, *p* < 0.001; hypomethylated: OR = 2.19, 95% CI: 1.94–2.46, *p* < 0.001; Fisher’s exact test).

To further investigate conserved regulatory DNA methylation alterations, we restricted the analysis to CpGs located within promoter or enhancer regions of the respective species. This identified 86 genes that were consistently associated with hypomethylation in regulatory regions in both human and murine BL as compared to the respective gcBC (Figure 3a, Supplementary Table S6). STRING network analysis of these genes revealed interactions among proteins involved in intracellular signaling, GTPase regulation, cytoskeletal organization, and B-cell-associated processes (Figure 3b).

Functional enrichment analysis of the 86 conserved hypomethylated regulatory genes identified significant enrichment of the Gene Ontology Biological Process term regulation of intracellular signal transduction (e.g., *GNA13*, *RASA3*, *RASAL1*, *SPRED2*, *SGK1*; FDR = 0.0007; OR = 7.72) (Supplementary Figure S3a). Consistent with this finding, KEGG pathway analysis identified significant enrichment of the sphingolipid signaling pathway (e.g., *PRKCE*, *PRKCA*, *PIK3R2*, *FYN*, *PPP2R5C*; FDR = 0.010; OR = 10.12). In addition, the conserved gene set included several genes with established roles in B-cell biology and transcriptional regulation, including *FOXO1*, *BACH2*, and *NR3C1*. Several additional GO and KEGG terms showed nominal enrichment but did not remain significant after multiple-testing correction.

In addition, 267 genes showed conserved hypermethylation at poised promoter regions in both human and murine BL (Supplementary Table S6). Enrichment analysis of these genes revealed significant enrichment for SUZ12 binding (Supplementary Figure S3b), indicating preferential association with Polycomb/PRC2-regulated genomic regions.

Together, these findings demonstrate that conserved DNA methylation alterations in human and murine BL preferentially affect regulatory chromatin states, with hypomethylation associated with genes involved in intracellular signaling and hypermethylation preferentially affecting poised promoter regions associated with Polycomb/PRC2 regulation.

## 4. Discussion

In this study, we asked whether DNA methylation alterations associated with human sporadic EBV-negative BL are conserved in a MYC/PI3K-driven murine model. Across species, we identified several shared features of the BL-associated methylation landscape. Both human and murine BL showed reduced global DNA methylation and distinct methylation profiles compared with gcBCs, as previously described by Kretzmer et al. [11]. More importantly, differential methylation showed a conserved chromatin-state pattern, with hypomethylation preferentially occurring at enhancers and hypermethylation at poised promoters. Together, these findings indicate that the MYC/PI3K-driven mouse model recapitulates regulatory features of the DNA methylation landscape observed in human EBV-negative BL.

The preferential hypomethylation of enhancers in both species points to a conserved involvement of regulatory elements in BL-associated epigenetic remodeling. Enhancers undergo extensive DNA methylation changes during gcBC differentiation and malignant transformation, reflecting the dynamic regulatory state of these cells [16]. The conservation of enhancer hypomethylation between human and murine BL therefore suggests that alterations of regulatory elements represent a shared feature of BL biology. In contrast, hypermethylation was strongly enriched at poised promoters in both species. The corresponding conserved gene set was also enriched for SUZ12 binding, consistent with an association with Polycomb/PRC2-regulated genomic regions. This finding is in line with previous observations linking cancer-associated DNA methylation to Polycomb-associated regulatory regions [38,39,40,41].

The strongest evidence for conserved biological mechanisms came from the cross-species analysis of regulatory genes. Among the 86 genes consistently hypomethylated in promoter or enhancer regions in human and murine BL, functional enrichment highlighted intracellular signal transduction and sphingolipid signaling. The gene set included several regulators of GTPase and kinase signaling, including *GNA13*, *RASA3*, *RASAL1*, *SPRED2*, *PIK3R2*, *PRKCA*, *PRKCE*, *FYN*, and *SGK1*. This finding is consistent with previous work by Kretzmer et al., who identified differentially methylated regions in BL associated with transcriptional regulation and recurrently mutated genes, supporting a link between DNA methylation and molecular pathways involved in BL pathogenesis [11]. Several genes in our conserved set also have established roles in gcBC biology and BL, including *GNA13*, *FOXO1*, and *BACH2*. In particular, *GNA13* regulates gcBC positioning and RHO-dependent signaling and is recurrently altered in human BL [42,43,44]. *FOXO1* is a key regulator of the germinal center dark-zone program and has an established role in BL, where nuclear FOXO1 promotes proliferation and survival and is recurrently mutated in both sporadic and endemic BL [26,45,46,47]. *BACH2* is an important regulator of gcBC survival and differentiation and cooperates with BCL6 to maintain the germinal center transcriptional program [48,49]. The convergence of these genes and pathways across species suggests that regulatory DNA methylation alterations may contribute to the epigenetic modulation of signaling pathways central to BL biology.

Among the conserved regulatory genes, *NR3C1*, encoding the glucocorticoid receptor, is of particular interest. *NR3C1* was hypomethylated in regulatory regions in both human and murine BL. Glucocorticoids are established components of treatment regimens for BL, and recent work has shown that glucocorticoid receptor signaling can inhibit oncogenic B-cell receptor signaling in BL and other aggressive B-cell lymphomas [50,51]. The conserved regulatory hypomethylation of *NR3C1* therefore provides a potential link between the epigenetic landscape of BL and glucocorticoid receptor signaling. Whether this methylation pattern influences *NR3C1*-mediated glucocorticoid responsiveness, or treatment sensitivity remains to be determined.

Several potential sources of biological variation should also be considered when interpreting the methylation patterns observed in the present study. Age- and tissue-specific DNA methylation patterns represent potential sources of biological variation in both human and murine samples. These factors were not specifically controlled for in the present study, as they were beyond the primary scope of the cross-species analysis. However, given the distinct biological context and the overall separation of BL from control samples, we consider them unlikely to substantially affect the main findings.

An important limitation of the cross-species comparison is that individual CpG sites could not be directly compared because human and murine DNA methylation was profiled using species-specific arrays. The comparison was therefore performed at the gene level based on conserved orthologs. Of the 2089 hypermethylated and 1681 hypomethylated murine genes with human orthologs, 695 and 512, respectively, showed concordant methylation changes in human BL. Thus, the MYC/PI3K model does not recapitulate the human BL methylome in a one-to-one manner, and the conserved alterations represent only a subset of the methylation changes observed in either species. Species-specific differences in genome organization and B-cell biology may contribute to these differences. Nevertheless, the conservation of specific regulatory methylation patterns indicates that the model captures selected epigenetic features relevant to BL biology.

## 5. Conclusions

The findings of this comparative cross-species DNA methylation study extend the molecular characterization of the MYC/PI3K mouse model to the epigenetic level. Notably, it demonstrates that key features of the regulatory DNA methylation landscape are conserved between human and murine BL. The identified genes and pathways provide a framework for further investigating the contribution of epigenetic regulation to BL biology and support the use of this model for studying conserved molecular mechanisms of MYC-driven lymphomagenesis.

## Data Availability

DNA methylome data produced in this study are available at GEO under accession number GSE347914.

## Supplementary Materials

The following supporting information can be downloaded at https://www.mdpi.com/article/10.3390/cancers18203408/s1, Figure S1. Principal component analysis (PCA) of murine samples based on the 10,000 most variable CpG sites; Figure S2. Copy number variation analysis in human and murine Burkitt lymphoma; Figure S3. Functional enrichment analysis of conserved differentially methylated genes; Table S1. Human samples used in this study; Table S2: Murine samples used in this study; Table S3. Differentially methylated CpGs in human EBV-negative Burkitt lymphoma; Table S4. Differentially methylated CpGs in the MYC/PI3K-driven murine Burkitt lymphoma model; Table S5. Gene lists underlying the cross-species overlap analysis shown in Figure 3; Table S6. Conserved differentially methylated genes in human and murine Burkitt lymphoma.

## Abbreviations

The following abbreviations are used in this manuscript:

BL	Burkitt lymphoma
EBV	Epstein–BarrVirus
gcBC	Germinal center B-cell
