# Supplementary material for: Conserved Regulatory DNA Methylation in Human and MYC/PI3K-Driven Murine EBV-Negative Burkitt Lymphoma
Source: Cancers (Basel). 2026 Oct 21;18(20):3408. doi: 10.3390/cancers18203408 (PMC13702466; doi:10.3390/cancers18203408)
Supplement: Supplementary file 1 [file cancers-18-03408-s001.zip › Figures_BL_Cancers_Supplementary.pdf]

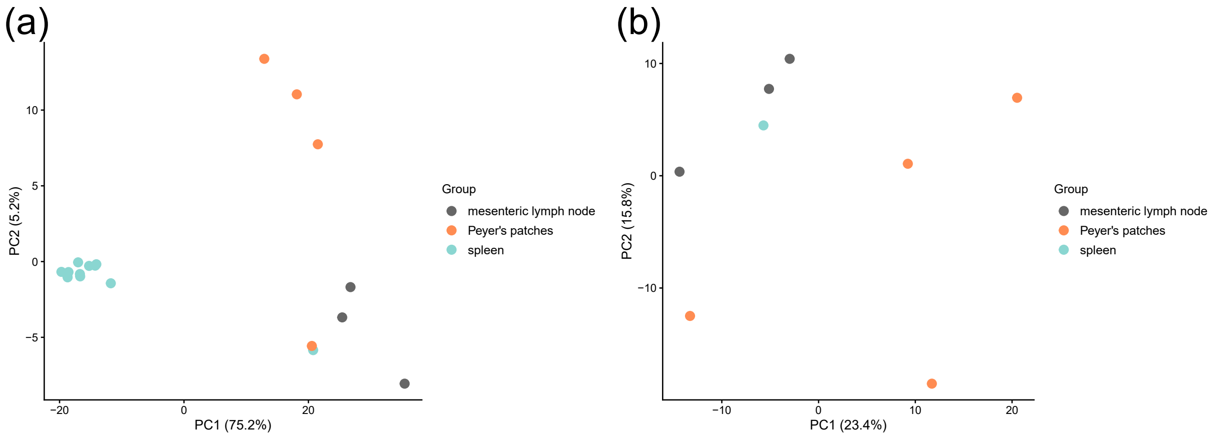

Supplementary Figure S1: Principal component analysis (PCA) of murine samples based on the 10,000 most variable CpG sites. (a) PCA of all murine samples, including MYC/PI3K-driven lymphoma samples and non-transformed controls. **(b)** PCA restricted to MYC/PI3K-driven lymphoma samples, annotated by tissue of origin.

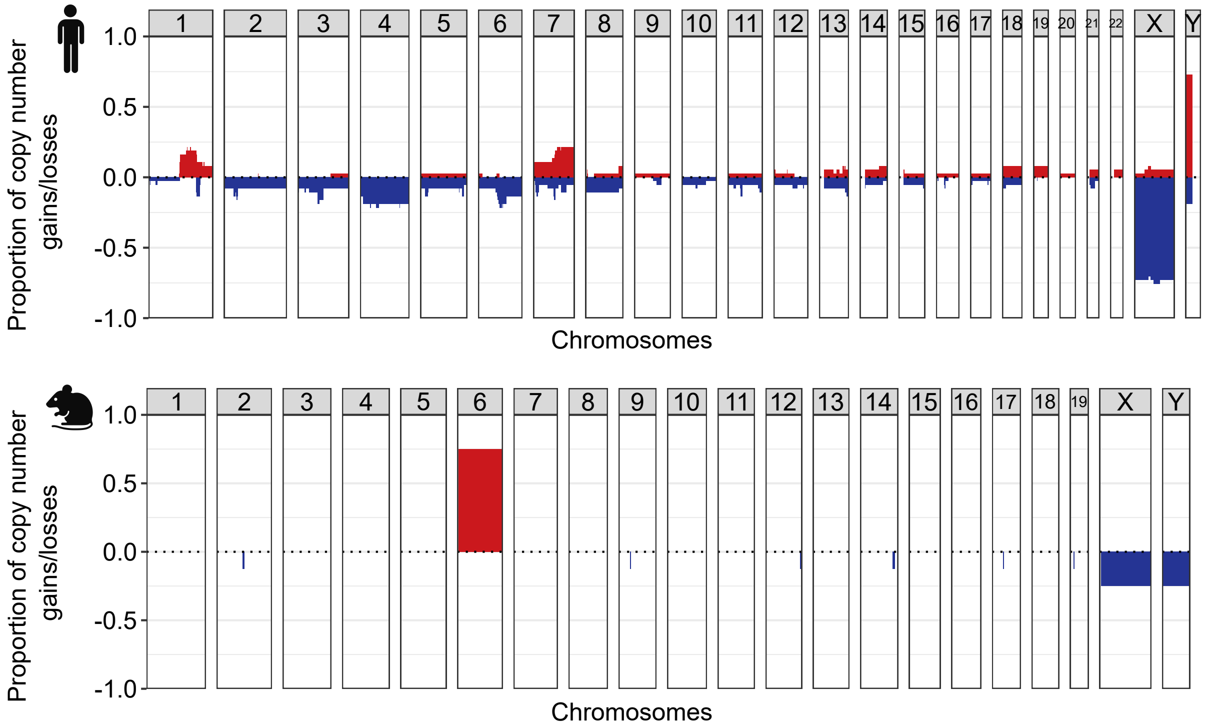

Supplementary Figure S2: Copy number variation analysis in human and murine Burkitt lymphoma. Frequency plots summarizing copy number alterations detected across all samples included in the study. The upper panel shows the frequency of copy number gains and losses across the human Burkitt lymphoma samples, and the lower panel shows the corresponding analysis for the murine Burkitt lymphoma samples. The x-axis indicates genomic position, and the y-axis represents the proportion of samples harboring a copy number alteration at each genomic region.

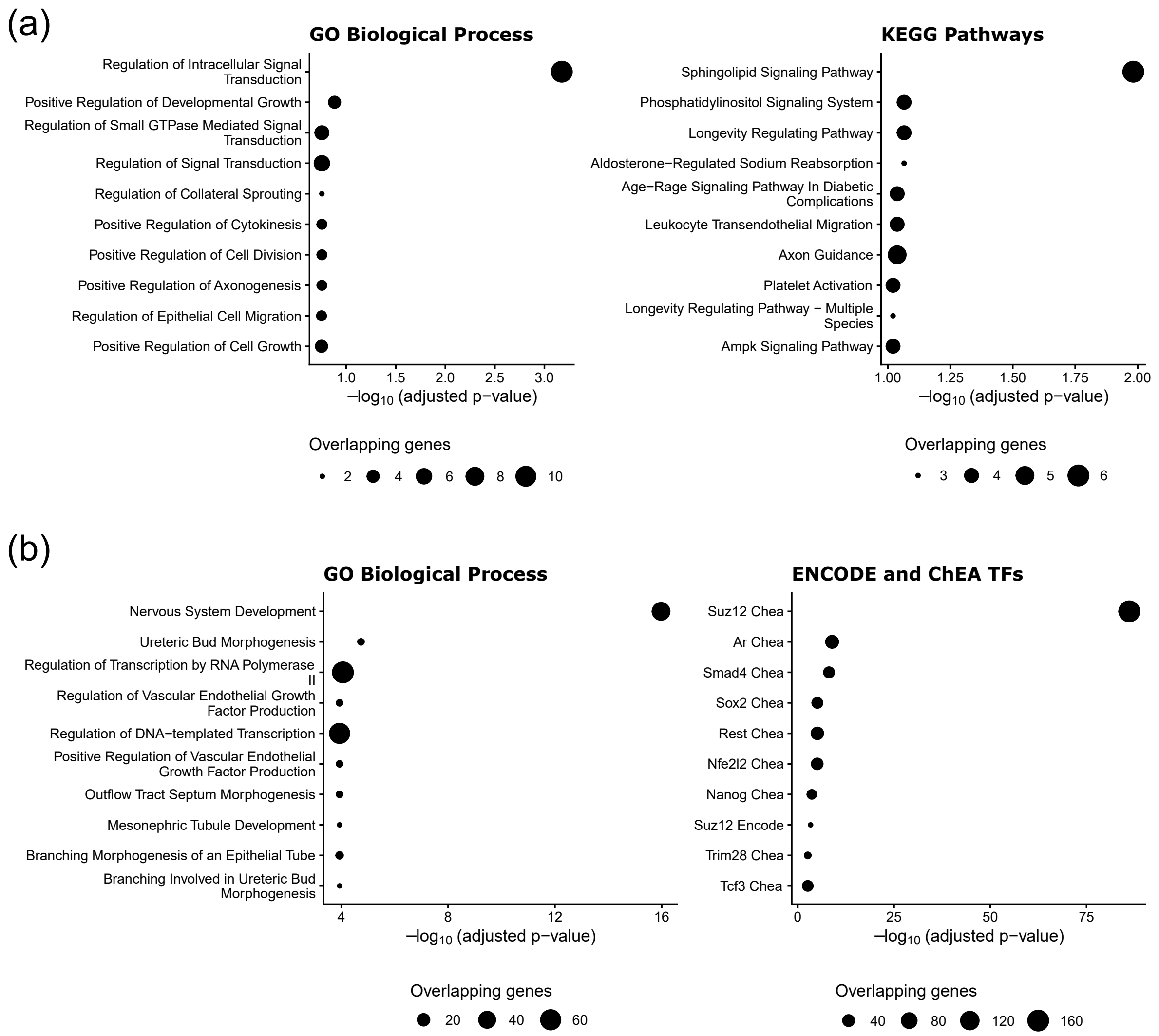

Supplementary Figure S3. Functional enrichment analysis of conserved differentially methylated genes. (a) Gene Ontology Biological Process (GO BP) and KEGG pathway enrichment analysis of the 86 genes hypomethylated within regulatory regions (enhancers or promoters) in both human and murine Burkitt lymphoma. **(b)** GO BP, ENCODE and ChEA transcription factor enrichment analysis of the 267 genes hypermethylated at poised promoter regions in both human and murine Burkitt lymphoma. For each analysis, the top 10 most significantly enriched terms are shown, ranked by adjusted P-value.
